# Moving from information and collaboration to action: report from the 4th international dog health workshop, Windsor in May 2019

**DOI:** 10.1186/s40575-020-00083-x

**Published:** 2020-05-07

**Authors:** Camilla L. Pegram, Brenda N. Bonnett, Helena Skarp, Gareth Arnott, Hannah James, Åke Hedhammar, Gregoire Leroy, Aimée Llewellyn-Zaidi, Ian J. Seath, Dan G. O’Neill

**Affiliations:** 1 Pathobiology and Population Sciences, The Royal Veterinary College, Hawkshead Lane, North Mymms, Hatfield, Herts AL9 7TA UK; 2 B Bonnett Consulting, Georgian Bluffs, Ontario Canada; 3 International Partnership for Dogs, c/o Svenska Kennelklubben, Box 771, SE-19127 Sollentuna, Sweden; 4 Svenska Kennelklubben, Box 771, SE-191 27 Sollentuna, Sweden; 5 School of Biological Sciences, Queen’s University Belfast, 19 Chlorine Gardens, Belfast, Northern Ireland BT9 5DL UK; 6 Canine Health & Welfare, The Kennel Club, London, W1J 8AB UK; 7 Department of Clinical Sciences, Swedish University of Agricultural Sciences, Uppsala, Sweden; 8 Université Paris-Saclay, Inrae, AgroParisTech, UMR GABI, 78350 Jouy-en-Josas, France; 9 Finch Research and Communications, Woodburn, Oregon USA; 10 Chairman Dachshund Breed Council UK, London, UK

**Keywords:** Welfare, IPFD, DogWellNet, Exaggeration, Extreme, Behaviour, Genetics, Genetic testing, Health, International

## Abstract

**Background:**

Dogs are the most popular mammal kept as a companion animal globally. Positive human-dog relationships can benefit both the human owners as well as the dogs. However, popularity as a companion animal species does not universally benefit dogs in reverse. Breed-related health problems in dogs have received increasing attention over the last decade, sparking increased concerns for dog welfare across many stakeholders. Progress towards improved welfare requires meaningful collaboration between all those working in dog health, science and welfare. The International Partnership for Dogs (IPFD), together with an alternating host organisation, holds biennial meetings called the International Dog Health Workshops (IDHW). The IPFD 4th IDHW was hosted by the UK Kennel Club in Windsor, UK in May 2019. With the aim of encouraging international and multi-stakeholder collaborations that are effective and ongoing, the 4th IDHW 2019 provided a forum to identify specific needs and actions that could improve health, well-being and welfare in dogs, building on outcomes and evaluating actions of previous IDHWs.

**Results:**

The workshop included 126 decision-leaders from 16 countries and was structured around five key themes identified as needing international, multi-stakeholder attention. These included the concept of “breed”, supply and demand, breed-specific strategies for health and breeding, genetic testing and extreme conformations. The review of progress made since the 3rd IDHW 2017 and the comprehensive lists of actions agreed upon during the current meeting suggest that movement from information and collaboration to action has been achieved. Working groups with specific tasks were identified and many plan to continue to communicate through forum communities on DogWellNet.com.

**Conclusions:**

The IDHW provides a forum for formal and informal discussion between relevant groups so that key dog health and welfare issues can be identified and defined, and plans can be agreed for effective actions to address them. The 3rd IDHW 2017 resulted in a number of significant outcomes. New and continuing actions were laid down at the 4th IDHW 2019, which will be re-evaluated at the 5th IDHW facilitating continual progress.

## Plain English summary

Dogs are popular companion animals worldwide. There are many advantages to dog ownership, however the popularity of dogs as companion animals does not always benefit dogs. Breed-related health problems in dogs have received increasing attention over the last decade, sparking increased concerns for dog welfare across many stakeholders. Progress towards improved welfare requires meaningful collaboration between all those working in dog health, science and welfare. This report aims to outline the structure, goals and outcomes of a large meeting held in the UK in May 2019 and assess progress made since the previous meeting in 2017.

The International Dog Health Workshops provide a forum to identify specific needs and actions that could improve health, well-being and welfare in dogs. The 4th International Dog Health Workshop 2019 included 126 decision-leaders from 16 countries and was structured around five important issues facing those who work to improve dog health. These included the concept of “breed”, supply and demand, individualised breed-specific strategies for health and breeding, genetic testing and extreme conformations. As at previous International Dog Health Workshops, the conference followed a framework with plenary sessions, facilitated breakout sessions, and allowed liberal time for networking.

Participants attending the workshops continue to be diverse and there are now well-recognised links between different stakeholders, making sustained action possible. Despite the diversity of participants, there are groups that could strengthen future discussions, such as breed health co-ordinators who play a pivotal role in positively influencing breeding decisions.

Significant progress has been made since the 3rd International Dog Health Workshop, although where the level of progress made had not met expectations this was highlighted and addressed. New and continuing actions were laid down at the 4th International Dog Health Workshop, which will be re-evaluated at the next workshop in 2021.

## Background

### Need for an international, multi-stakeholder approach to dog health and welfare

Dogs were first domesticated over 10,000 years ago and were originally used as working animals, only recently making the shift towards a predominantly companion animal role [1]. Dogs are the most popular mammal kept as a companion animal globally, largely stemming from the deep human-canine bond, with a recent survey across 22 countries estimating that 33% of people live with a dog [2].

Positive human-dog relationships can lead to physiological and emotional changes that benefit both the human owners as well as the dogs [3]. Benefits to humans from dog ownership (compared with non-dog ownership) include increased levels of physical activity [4], better sleep patterns [5], reduced risk of cardiovascular disease [6] and lower mortality [7]. Beyond physical health benefits, dogs can provide important social and emotional support [8].

For these reasons, it is widely accepted that dog ownership should be encouraged [9]. However, popularity as a companion animal species does not universally benefit dogs in reverse. For example, breeding practices that prioritise aesthetic appeal over healthy conformation can increase the expression of inherited defects and thus compromise the health and welfare of many breeds [10]. Breed-related health problems in dogs have received increasing attention over the last decade, sparking increased concerns for dog welfare across many stakeholders [11]. Responsibility for controlling such health problems has variously been directed at dog breeders, kennel clubs, the veterinary profession and consumers [12–17]. A report following the 3rd IDHW in Paris emphasised that, instead of any single group being accountable, all of these stakeholders, as well as other influencers such as the media and celebrities, should share overall responsibility for dog welfare because each plays an important but differing role in a complex interplay [18]. No single stakeholder group alone can resolve these issues; progress towards improved welfare requires meaningful collaboration between all those working in dog health, science and welfare.

### The need addressed

The first IDHW was held in June 2012 in Stockholm, Sweden, where the need for an international platform for collaborative efforts was stressed, and this triggered the development of the International Partnership for Dogs (IPFD), a non-profit organisation initiated by several national Kennel Clubs and other stakeholders in dog health. The aim of IPFD is to facilitate collaboration and sharing of resources to enhance the health, well-being and welfare of pedigreed dogs and all dogs worldwide; and to support human-dog interactions [18]. DogWellNet.com was subsequently launched as the internet platform of the IPFD [19]. The second and third IDHWs were organised by the IPFD together with a local host kennel club: Dortmund, Germany in 2015 (hosted by the German Kennel Club) and Paris, France in 2017 (hosted by the French Kennel Club). From May 30th – June 1st, 2019, the IPFD 4th IDHW [20] was hosted by the UK Kennel Club in Windsor, UK with Royal Canin as the major sponsor. With the aim of encouraging international and multi-stakeholder collaborations that are effective and ongoing, the 4th IDHW 2019 provided a forum to identify specific needs and actions that could improve health, well-being and welfare in dogs, building on outcomes of previous IDHWs.

The 4th IDHW 2019 meeting was formatted around 5 key themes covering issues that regularly feature as discussion points in relation to breed-related health in dogs. Three of these five themes previously featured in the 3rd IDHW 2017, namely Breed-specific Health Strategies, Exaggerations and Extremes in Dog Conformation and Genetic Testing for dogs, and were repeated in order to promote further development of the action plans that were previously defined. The Concept of “Breed” and Supply and Demand were new themes for 2019.

Understanding the concept of breed, namely what defines a breed and how this concept influences health and welfare of dogs, is essential to implement effective control strategies [21]. Health control measures regularly take breed-specific approaches, since individual breeds often have their own health concerns and priorities as well as their own frameworks for data collection and control measure implementation [22]. Individual breed-specific strategies for health and breeding are needed but may vary by country, and therefore sharing tools to support the work of breed clubs is beneficial [1, 14, 23]. The UK Kennel Club (KC) Breed Health and Conservation Plans (BHCPs), for example, aim to identify health concerns for individual breeds using evidence-based criteria from which balanced breeding decisions can be generated [24]. Efforts to understand and address health and welfare problems in dogs are complicated by issues around the varied means of sourcing puppies. Demand for dogs remains high and in many countries the majority of apparently purebred dogs are thought to come from commercial breeders who are not registered with relevant kennel or breed clubs and therefore may fall outside the normal influences, controls and regulations of such bodies [25]. Welfare levels and outcomes may vary depending on sourcing, with puppies obtained from pet stores predisposed to potential owner-directed aggression as adults [26]. There are continual advancements in genetic testing for dogs, however this can make decision-making on appropriate tests more challenging [1, 27]. The Harmonization of Genetic Testing for Dogs (HGTD) provides guidance on the appropriate selection and optimal use of genetic tests in dogs [28]. And finally, exaggerations and extremes in dog conformation can negatively impact the health and welfare of individual dogs. Whilst national [29–31] and international efforts [32–34] have been implemented to understand and mitigate these issues, education and communication are key to promoting desirable human behaviour change [35, 36]. It is becoming increasingly clear that many of the problems of dogs at a population level are really issues related to human perceptions, and almost all are influenced by human decisions. Previous evidence has shown that higher levels of health and behaviour problems in certain breeds are positively associated with a closer owner-dog relationship [16]. Owners of brachycephalic dogs are often aware of their breed’s health issues, but a high proportion still believe their own dog has very good health or the best health possible [37]. Understanding the best strategies to positively influence human behaviour change are critical to improve the health and welfare of dogs [38].

A previous publication has described the background, process and action plan from the 3rd IDHW [18]. The current report builds on this with a focus, as well, on the next planned meeting, the 5th IDHW 2021. This report aims to outline the structure, goals and outcomes of this meeting and to assess progress made since the 3rd IDHW 2017.

## Methods

### Meeting format

As for all previous IDHWs, the programme for the 4th in 2019 focused on key themes based around challenges identified as needing international, multi-stakeholder attention (Table 1). The 4th IDHW was organised following a similar format to previous meetings, which has been detailed in the report published on the 3rd IDHW in 2017 [18]. In total, 126 participants from 16 countries attended the 4th IDHW 2019, comprising decision-leaders from most major stakeholder groups in dog health and welfare. The attendees were diverse and included breeders, members of breed club health committees, kennel clubs, breeding advisors, veterinarians, educators, researchers, geneticists, behavioural specialists, regulators, welfare organisations, industry, media, health campaigners, dog owners and show judges. It was noteworthy, however, that there were few breed health co-ordinators present and therefore efforts should be made to facilitate attendance by this key stakeholder group at future meetings.

**Table 1:** Six overall themes for the 4th International Dog Health Workshop in 2019 in Windsor, UK

Theme	Session leader(s) (number of participants)
The Concept of “Breed”	Helena Skarp, Sweden; Astrid Indrebo, Norway [14]
Supply and Demand	Gareth Arnott, Nothern Ireland; Sarah Ross, Germany; James Stephens, Ireland; Candace Croney, USA [11]
Breed-Specific Health Strategies	Ian Seath, UK; Gregoire Leroy, France [18]
Genetic Testing for Dogs	Aimee Llewellyn-Zaidi, USA; Brenda Bonnett, Canada; Claire Wade, Australia; Sue Pearce-Kelling, USA [27]
Exaggerations and Extremes in Dog Conformation	Ake Hedhammar, Sweden; Tamzin Furtado, UK; Pekka Olson, Sweden [22]

As at previous IDHWs, plenary presentations from international experts on the morning of the first full day were followed by breakout sessions for each theme spread over the 2 days and interspersed with two sharing-sessions in plenum [18]. In advance of the conference, participants were provided with information, including surveys on topics relevant to their specific themes, in order to focus discussion and activities both during and after the meeting. The use of online polling within the plenary and summary sessions gathered and shared common opinions. The meeting went beyond mere discussion, with the aim to generate meaningful and concrete outcomes. Pre- and post-meeting resources and material for the 4th IDHW 2019 are available on DogWellNet.com [20]. This paper explores progress made since the 3rd IDHW 2017 before summarising the discussions, recommendations and actions identified and committed to by participants during the 4th IDHW 2019.

## Results

### The 3rd IDHW 2017: review of work themes and outcomes to date

#### Breed-specific health strategies: needs and opportunities; innovations, nationally and internationally

Since the 3rd IDHW, resources related to breed-specific health strategies have been developed, refined and added to DogWellNet.com [39]. This site now shares exemplars of strategy documents from many breeds, for example, the Swedish Breeding Strategy (RAS) [40] and Finnish Breeding Strategy (JTO) [41] formats and Breed Health and Conservation Plans (BHCPs) [42] from the UK. Templates for these are also now available, together with a PowerPoint presentation summarising the range of components that might be expected in a strategy. The DogWellNet site also includes guidance documents, such as the UK KC’s Breed Health Strategy Guide [43], and a number of blog posts on the theory and practice of strategy development and implementation. International discussions using DogWellNet online forums have been initiated, which review evidence and critique published papers of relevance to the participants’ breed-specific strategies. This theme was carried forward to the 4th IDHW 2019; see further information in the relevant section for the 4th IDHW 2019 below.

#### Exaggerations and extremes in dog conformation: health, welfare and breeding considerations; latest national and international efforts

This theme was carried forward to the 4th IDHW 2019 and therefore progress is included in the relevant section for the 4th IDHW 2019 below.

#### Education and communication: how can international collaboration improve education and communication within and across stakeholder groups [especially between veterinarians and breeders]; using the example of antimicrobial resistance

The emergence and expansion of antimicrobial resistance (AMR) has been widely documented and challenges current antimicrobial therapy protocols [44, 45]. A notable outcome from this theme was the funding of a veterinary student at Ohio State University to create educational material during the summer of 2017. Resources from this project included articles, blogs and educational videos for veterinary students. These, and an expanding collection of other resources are found on the Antimicrobial Resistance Resources Index page on DogWellNet.com [46].

#### Behaviour and welfare: how can we better integrate actions to address issues in welfare, behaviour and health in breeding and raising dogs?

This theme featured in each of the first 3 IDHWs because optimal behavioural development of dogs is critical to facilitate their life as companion animals within human homes. Since the 3rd IDHW 2017, a poster entitled “Puppy Socialization in 5 Points” has been produced which is available on DogWellNet.com and was shared internationally as a positive marketing message [47].

#### IPFD harmonization of genetic testing for dogs: an international, multi-stakeholder initiative to address selection, evaluation and application of genetic testing

This theme was carried forward to the 4th IDHW 2019 and therefore progress is included in the relevant section for the 4th IDHW 2019 below.

#### Show me the numbers: integrating information from various sources for prevalence, risks and other population-level information; latest national and international strategies to collect data and disseminate information

This theme was prioritised because data-deficiencies are widely acknowledged to constrain improvement in companion animal health and welfare [48]. Indeed, the need for improved quantitative data was identified in each of the other five themes at the 3rd IDHW 2017 as a critical limitation for successful progress. Substantial progress has been made in the intervening time to the 4th IDHW 2019 towards achieving the planned actions to encourage development of programmes that apply veterinary clinical data for research. VetCompass in the UK now includes over 1800 veterinary practices (> 30% of UK practices) and has published 67 peer-reviewed papers [49]. Savsnet, also in the UK, includes over 500 veterinary clinics and additionally collects data from veterinary pathology laboratories [50]. PETscan in the Netherlands has continued to develop its systems for the collection and analysis of veterinary clinical data [51]. VetCompass Australia has now become firmly established with several research projects underway [52]. Also priortised at the 3rd IDHW was movement towards open access publication to encourage wider disemination of research findings and there is some evidence that research papers are increasingly likely to be published as open access since then [37, 53, 54]. There has also been evidence of increased research output on two key areas identified as priorities at the 3rd IDHW 2017: disease prevalence/incidence, risk factors, and geographic spread [53, 55–58] and also quality-of-life and end-of-life data [59, 60].

### The 4th IDHW 2019: work themes and actions planned

As described above, the 4th IDHW 2019 was structured around 5 key themes that were identified to need international, collaborative attention and actions to improve breed-related health in dogs (Table 1). Each theme is described below with information provided on the discussions that took place and the actions proposed by participants.

#### The concept of “breed”

In the mid-nineteenth century dog breeds, as we use the term today, were invented and developed [61, 62]. The development of breeds meant that the variation in phenotype (and consequently in genotype) that was present up to that time was to a great degree, either lost or divided into different breeds [63]. Breeds were often created using small numbers of founder animals with genetic bottlenecks as a result [64]. Selection became focused on establishing homogeny within breeds and also distinction from other related breeds. Breed studbooks were largely closed and crossbreeding/interbreeding was, with few exceptions, banned within pedigree registered breeds [65].

This, in a historical context, new approach to dog breeding has resulted in relatively low genetic variation within breeds, even for numerically large breeds [66, 67]. Low genetic variation and inbreeding has been associated with reduced health and fertility and can be a limiting factor when planning actions to breed for better health [68].

Breeding goals for behaviour, mental traits, working ability and conformation continue to change over time. New breeds continue to be recognised by canine organisations [69]. New breeds are often, in fact, divisions of pre-existing breeds based on differing breeding goals within breed (e.g. different coat colours/coat types or different sizes), reflecting varieties within a breed, rather than true breeds. Notwithstanding, the number of breeds in the world continues to increase over time [70]. Altered breeding goals regarding conformation often result from changed fashion in the show ring and such changes can sometimes result in health problems due to exaggerated features, such as in the Rottweiler [71].

In their respective plenary talks, available on DogWellNet.com [72], Helena Skarp, “The Concept of Breed: Past, Present-and Future?”, Gregoire Leroy “Definitions of Breeds Across Species and Countries: Crosscutting Concepts” and Peter Friedrich, “On the origin of Breeds” discussed the situations described above and posed questions for the attendees to consider. Collectively, these talks emphasised that the breed concept is multi-dimensional, complex, changes over time and is subject to the whims, attitudes and beliefs of humans [73]. Ms. Skarp challenged the group to consider whether the current definition and regulation of ‘breed’ is compatible with, and adequate for, securing a sustainable future for the health and welfare of breeds. Dr. Leroy highlighted that increasing impacts of health concerns as well as scientific advances in genomics were impacting the understanding of ‘breed’ and that issues of breed governance were also evolving. As he discussed in his article referenced above, Prof. Friedrich showed compelling examples of how trends in fashion within the dog world were having major impacts not only on appearance but on the very nature and stability of dogs and breeds.

##### Discussions

Breed conservation, in the sense of maintaining a specific phenotype and genepool, versus breed development, i.e. allowing a population to change and evolve, was discussed. One conclusion was that in an in situ context, the genepool of a breed is bound to evolve, which can be considered positive or negative depending on the perspective. The group stated that, except for traits connected with health problems, breed development should be more flexible for the public/fashion demand and that focus should be on dog health. For example, many breeds may now primarily be bred as companions, rather than for their original functional purpose [74]; this is allowable, as long as the changes in appearance or functionality from this trend do not create or exacerbate health problems.

Breeds that regularly feature individuals born with anatomic features which are (more or less) genetically incompatible with the breed standard (e.g. ridgeless dogs in ridgeback breeds, coated dogs in hairless breeds) were discussed. The group suggested that varieties or individual dogs lacking such breed standard traits should be fully accepted for breeding and showing (e.g. both ridgeback and ridgeless) and that breeding between such varieties should be allowed. Both in the plenary sessions and breakout sessions, it was agreed that the increased number of varieties that are maintained as distinct entities within many registries may pose risks for maintaining intra population genetic variability. The group stated that the increasing number of breeds in itself would not be a problem provided that interbreeding, performed in a controlled way, is permitted between related breeds and/or breeds with common breeding goals.

Two methods to increase genetic variation within breeds were suggested: 1. open studbooks between related breeds and/or breeds with common breeding goals and/or admittance of individuals without pedigree to enter the studbook providing specific conditions (e.g. phenotype corresponding to the breed standard) and 2. cross-breeding projects. In each case, it was specified that these must be well-planned strategies (Table 2).

**Table 2:** Suggested methods to increase genetic variation within breeds and proposed implementation

Type of method	When to use	Breeds to be used for interbreeding/cross- breeding	Time frame
**Open studbooks/interbreeding**	Recommended for all breeds	Closely related breeds and/or breeds with a common breeding goal	Long term
**Cross-breeding projects; i.e. well-designed, with clear goals and outcomes; controlled and monitored.**	To increase genetic diversity and/or introduce/improve desired genetic traits	Breeds possessing the desired trait	Limited/specified time period

The Fédération Cynologique International (FCI) list of crossing between breeds and varieties [75] was considered a good starting point and the group stated a wish for this concept to be explored and potentially expanded to create an environment of permission for national KCs to employ more open registries. The Finnish KC has developed protocols for cross-breeding programmes [76].

To move forward on cross-breeding projects, the following suggestions were made:

- Use the data from cross-breeding projects (particularly aimed at health or hybrid vigour) to promote further activity/development. If more data are needed, we should collect such data.
- Create guidelines, a quality standard and outline methodology for cross-breeding projects.
- To achieve an improvement of the more important positive aspects, (e.g. health and/or mentality and/or working ability) we may have to tolerate some negative aspects (e.G. *minor* conformational change).

##### Goals and strategies

The primary focus of all recommendations made by the group is improved health and welfare of all dogs.

*Goals* Long term:

- Open studbooks between related breeds and/or breeds with common breeding goals in a controlled way.
- Cross-breeding projects should be used when a health or welfare problem cannot be solved within breed.
- KCs registries should be open for all dogs, not only pedigreed dogs.

Short term:

- Create a positive attitude to the concept of more open studbooks.
- Encourage the development of cross-breeding projects.
- Encourage KCs to record all dogs in a database (in a separate registry, not the stud book registry used for pedigree dogs).

*Strategies*

- Collaborating with breed clubs and breeders at an early stage.
- FCI Scientific and Standard Commission in cooperation with other kennel clubs should take on the task to develop guidelines to help kennel clubs and breed clubs on how to proceed with breeding between varieties and breeds.
- Consult and identify breed issues where the previously mentioned guidelines and methodologies should be used.

#### Supply and demand

The plenary talks included an overview by Dr. Brenda Bonnett; CEO of IPFD; Sarah Ross from Four Paws international animal welfare organisation who described issues and initiatives around the illegal online puppy trade in Europe; and Jim Stephens who described the Irish situation in terms of supply and demand. Prof. Candace Croney of Purdue University in the USA, gave an insightful talk on her work on welfare of dogs in commercial breeding establishments and creation of standards [77]. These talks are available on DogWellNet.com [72].

The breakout for this theme brought together 18 participants from a diverse range of backgrounds including; veterinarians, kennel clubs, breed clubs, welfare organisations, and academic researchers. This provided a breadth of expertise well-suited to discussing the complex and cross-cutting issues associated with the supply and demand of dogs. Despite substantial ethical concerns, consensus was reached to recognise that commercial breeders (see further definition below) currently have and will continue to have a role in the supply of dogs, with the debate having moved beyond calls for their total elimination. However, there was also agreement that important concerns remain regarding the welfare of dogs (both breeding animals and puppies) within these commercial breeding establishments, and that these should be addressed as a matter of priority [78]. A number of useful models for breeding regulation were discussed, e.g. from the Swedish Kennel Club and The UK Kennel Club [79].

Arising from these discussions, the group identified four goals:

1. A need to establish a framework for the “Sustainable supply of dogs”. Currently, opinions differ between stakeholders and individuals regarding what constitutes a commercial breeder, and it was acknowledged that the theme would benefit from clarifying and defining this aspect. The goal of “sustainability” in this context is aimed at recognising that dogs will be sourced from a range of origins depending on owner priorities. We need to better understand the numerical contributions of puppies from each of these sources which include; kennel club registered breeders, commercial breeders, hobby and ‘backyard’ breeders, animal shelters, rescue organisations and charities.
2. An urgent need to better understand the numbers and origins of pet dogs. Discussions during the theme revealed key knowledge gaps regarding pet dog population sizes and the numbers of puppies needed to supply existing demand. It was acknowledged that current figures are crude estimates, with a lack of accurate statistics. Continued and improved dog traceability schemes through registration and identification involving microchipping will be required to address this goal (see actions below).
3. To gain a better understanding of the role of the internet and social media in facilitating the supply and demand for dogs. All stakeholders of the theme recognised the importance of internet resources and social media for purchasing dogs but highlighted that there are a number of important knowledge gaps and areas of concern regarding this aspect.
4. To gain a global perspective on the existing legislation relating to the regulation of commercial dog breeding. At present legislation governing dog breeding varies between and within (USA variation between states) countries. Furthermore, the evidence base informing this type of legislation is frequently weak or absent. Collation of this information into a central database would enable identification of common themes, sources of variation, and areas in need of research to inform policy.

To achieve these goals, working groups were created, comprising individuals committed to undertake the following actions:

1. Compile an international database of legislation related to commercial dog breeding.
2. Continued engagement and lobbying on dog traceability and microchipping. This action recognises that while a number of countries already operate successful registration and identification schemes [80] such schemes are lacking or function inefficiently in other countries (e.g. the UK is limited by the existence of multiple databases from different microchipping companies). It was acknowledged that with the EU Animal Health Law applying from April 2021 there is an opportunity to make use of this law to improve traceability across the EU [81].
3. Research to inform knowledge gaps related to supply and demand including:

- A literature review to identify socialisation protocols that facilitate appropriate behavioural development.
- Continued research examining behaviour and welfare within commercial breeding establishments.
- Quantifying the economic value of dog breeding.
- Consumer behaviour analysis.
- Continued research on factors influencing relinquishment of dogs to animal shelters.

4. Social / online media engagement. This will include continued communication with online selling platforms to improve standards and levels of regulation.
5. Establish an education programme for relevant stakeholders (e.g. breeders, veterinarians, shelter staff, enforcement officers) to raise standards of welfare.
6. Facilitate attendance of commercial breeder representatives at the next IDHW. This acknowledges the importance of “Human Behaviour Change” research and the role of having all relevant stakeholders participate in discussions.

#### Breed-specific health strategies

Brenda Bonnett presented a plenary presentation highlighting the resources on and planned for DogWellNet.com while Ian Seath delivered an insightful presentation on strategic planning for breed health, in the context of the realities and challenges for breed clubs. The slides from these talks are available online [72].

In the breakout discussions, the group reviewed the challenges facing breed clubs in designing and implementing health strategies and explored how different stakeholders can collaborate to tackle these problems. A breed club’s overall approach and understanding of each health condition is critical, in that lack of engagement or misunderstanding of the relevance to breeding stock can have severe consequences when trying to improve the breed as a whole. In addition to this, trust and transparency between parties (e.g. kennel clubs, veterinarians, breeders & researchers) play a role in advancing health. Information can be incorrectly relayed to lay people, or owners may have concerns regarding the protection of their data. The sphere of influence of breed and kennel clubs can also differ greatly between breeds and countries [23]. Anecdotally, an overall decline in breed club membership internationally, and therefore influence, consequentially reduces the dissemination of vital health information. The group aimed to develop solutions to these specific hurdles, which can be implemented on an international level.

Participants of the group included veterinarians, geneticists, breed database developers, breed club members, Kennel Clubs and dog owners. The breed specific Irish Wolfhound Database [82] and Berner-Garde (a database of information about Bernese Mountain Dogs) [83] were presented to the group. These are platforms that assist breeders to make informed decisions when choosing a mate, as well as detailing trends in disease and genetic diversity. Ultimately, these examples highlighted the scope of achievement that is possible from international collaboration.

The group agreed that collaboration on an international level is a necessity to develop achievable health strategies, and that each stakeholder must tailor their approach dependent on the receiving audience. It was agreed that collaborative initiatives such as the IDHW allow development of strategies and solutions to common problems faced by the breeds.

With regard to misunderstanding amongst breeders of the need for health strategies and concerns of exposure within the breed community (e.g. assigning blame to certain individuals, attacks on social media), the importance of raising the profile and relevance of health issues was discussed. In livestock conservation or development programmes, it has been shown that breeders’ adoption of the strategies and interventions (or difficulties in adopting them) and the presence (or absence) of an intermediary person or structure may be critical in the success of such programmes [84]. The potential to use external bodies to reduce concerns over lack of transparency and to maintain confidentiality was felt to be a useful strategy. Furthermore, impressing health awareness on the public will help to engage less enthusiastic or apathetic breeders and encourage their participation in implementing health strategies. Similarly, open publication of health results puts an onus on non-compliant breeders, particularly with the current open culture seen on social media. The method of approach to breeders and owners was also raised, in that this should be addressed in a manner which does not blame the breeder, but rather asks how they perceive health and what they would like to address for the betterment of their breed. Prioritisation of health concerns to ascertain achievable goals is fundamental to the efficacy of strategies [36], and would also act to prevent alienation of breeders.

The use of multiple platforms to disseminate information amongst puppy buyers and breeders/owners is vital, for example developing online groups, breeder seminars and health days, and targeted advertisements (e.g. Google Ad grants). These platforms can also be used to monitor engagement of puppy buyers and owners.

Kennel Clubs are a suitable source of assistance in health strategies [24], in that they can target owners and breeders outside of club spheres of influence and often have in-house experts who can assist in defining manageable metrics, as well as analysis and data interpretation [23]. The latter factor also combats a further problem that breed clubs may face with regard to lack of scientific expertise within their breed, resulting in unrealistic expectations and potential bias of data. Providing feedback and data results to the entirety of breed representatives would also reduce perceived penalisation of individual breeders and ensure responsibility for the breed’s health is widespread among the community of breeders. The importance of raising awareness of currently available resources and templates was stressed, in that this can make the process much easier for breed clubs and improve the collection of data.

With regard to the reductions in breed club membership and the reduced breadth of influence (in part due to declining membership), the possibility of introducing subsidies, e.g. financial support from clubs or their foundations, to encourage owners to join was discussed, as well as collaborating internationally with other breed clubs, or merging clubs together to form parent clubs and councils. To further encourage participation in health programmes amongst breeders and owners, reward incentives could be a possible solution, recognising those that contribute throughout the health strategy, e.g. with certificates, subsidies, etc. Similarly, compensatory funds could be established for breed clubs, for use should a health problem become apparent in a puppy bred under a health strategy. This would act to emphasise the reduced risk of buying a puppy from a responsible breeder.

Actions agreed by the group included to:

1. Develop a landscape overview of how the national Kennel Clubs and breed clubs work and the sharing of responsibilities between these parties. This is to understand how these processes could be replicated in other countries and to allow understanding of the different options for implementation of health strategies.
2. Create a forum on DogWellNet.com whereby the group can share successful tactics used in dogs and other species – specifically with regard to genetic diversity, and develop tactics for engaging owners outside of breed communities.
3. Targeting the next generation of veterinarians (for example through veterinary student days and specific education) is an international effort that needs to be undertaken, to ensure information is disseminated in the most accurate manner and from as many routes as possible.
4. Build a formal implementation cycle for health strategies with applicable tools and resources [85], which can then be shared with the Kennel Clubs and breeds.

#### Genetic testing for dogs

This theme built on discussions from the 3rd IDHW 2017’s *IPFD Harmonization of Genetic Testing for Dogs* (HGTD) theme. Defining “good quality” genetic test providers (GTPs) and DNA testing, in the current absence of independent regulation, remains a challenge for dog owners, veterinary scientists, and breed/ kennel clubs. Since the 3rd IDHW 2017, the Harmonization of Genetic Testing (HGTD) project has continued to be developed as an open-access resource incorporating data on the genetic test providers (GTPs) including quality indicators and information on offered tests via DogWellNet.com. Comprehensive GTP data are held on 42 participating GTPs from 22 countries. Basic data are held on an additional 34 non-participant GTPs. In addition, there is collated information, including genes/mutations, clinical information, and breed-specific applications information on 302+ phenes/diseases. Alongside this database, there is further work underway or planned addressing a broad-spectrum of concerns regarding genetic testing (GT). Further developments include platforms for expert reviews of tests; supporting a proficiency testing scheme, genetic advice, and GT education. The model continues to depend on GTPs and multi-stakeholders participating voluntarily and, in some cases, financially.

The 32 theme participants included representatives from GTPs, kennel clubs/registration bodies and welfare groups, as well as veterinarians, geneticists and researchers, canine health campaigners, and owners/breeders, and discussions aimed to identify priorities and actions needed. General aims outlined in the plenary introduction [86] included: support consumers, and confidence in DNA testing; breeding practices to reduce or eliminate inherited diseases and promote healthy offspring; the further scientific understanding of inherited diseases; and reduce redundant world-wide efforts. Plenary presentations also included Prof. Claire Wade from the University of Sydney, discussing challenges in research and discovery that affect application [87]. Breakout session discussions addressed the need for independent evaluation of genetic tests, and a process to do so; defining levels of “validation”; addressing peer-review and publication issues, and how to provide genetic advice, especially via the IPFD Expert Panel development.

**Fig. 1:**
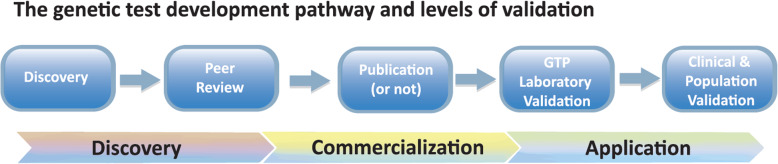
Validation Matrix: representation of the needed flow of different forms of genetic test “validation” from discovery to application in dog populations

The group identified five major action areas to address via the HGTD and for each, a working group was identified to move the projects forward:

1. **Launch of the IPFD Expert Panel** by which queries around individual tests and their application in breeds, as well as other technical and genetic counselling aspects could be addressed and shared with the public, will be further developed and beta-tested within 2019–20.
2. **Laboratory quality standards and technical proficiency** were discussed with particular reference to self-assessment and reports [88], and a working group was established including both commercial and academic GTPs to take this forward.
3. **Proficiency testing** is being explored in discussions with the International Society for Animal Genetics (ISAG); decisions made at the ISAG meeting in July 2019 are moving this forward.
4. **Development and use of a “validation” matrix/template** –Validation concerns, both at the technical/laboratory and clinical/population levels (particularly across breeds), proved to be a major concern among all participants. Both researchers and GTPs raised various issues with peer-reviewed publication (or lack thereof), and establishing a pre-publication review process is being explored. A preliminary model of the Validation Matrix was drafted and discussed (Fig. 1)
5. **Sustaining ongoing support of IPFD and HGTD** Is critical to most of the actions identified and engaging ongoing and additional funding sources will be explored.

Creation and discussion of the validation matrix by highly-qualified participants representing a broad cross-section of canine health and welfare stakeholders was critical in refining the working groups and resources needed in moving forward.

Across the theme, all prioritised actions will inter-relate and contribute to addressing issues in GT. While many concerns exist within the existing GT landscape, collective and collaborative actions are the best way forward to ensure that genetics and genomics can achieve their phenomenal potential to impact health in dogs and dog populations. Continued and expanding support of the IPFD and its various platforms was deemed as critical to underpin ongoing success.

#### Exaggerations and extremes in dog conformation

Extreme conformation has been defined as when an animal’s body shape, structure or appearance has the potential to result in negative impacts on their health and welfare [89]. Breeding decisions should aim to confer healthy or healthier conformational traits and this generally requires a move away from extreme conformation. There are now widespread calls to limit the current preference of dog owners for breeds with extreme conformation [90].

The exaggeration and extremes theme gathered 22 participants representing 8 countries. Continuing on from the 3rd IDHW in Paris, this theme focused on the brachycephalic issue and especially on Brachycephalic Obstructive Airway Syndrome (BOAS). This ensured that the 4th IDHW 2019 could move on from prioritising the main issues to discussing optimal actions, since this was a familiar topic for the majority of theme participants.

Despite the focus being on BOAS, the group agreed that a holistic view for an effective action plan was needed to ensure other problems associated with the brachycephalic phenotype or related to brachycephalic ownership were not neglected. The strong influence of human behaviour patterns on the current fascination of the public for breeds with extreme conformation was brought home in a fascinating plenary talk from Tamzin Furtado [72].

It was agreed that that the theme would have benefitted from increased representation from important groups, such as breeders, breed health coordinators and show judges of brachycephalic breeds; their absence, in spite of efforts to encourage their participation, was a serious limitation to progress. It was concluded that increased efforts must be made to engage representatives of these stakeholder groups at future meetings.

The discussions and conclusions from the session were:

1. The need for improved communication and change of perception

Despite many good actions to date, the group did not believe that the perception by the general public of brachycephalic breeds as ‘highly desirable breeds to own’ has been sufficiently altered. The importance of improved communication between involved stakeholders has been recognised at previous IDHWs and therefore the 4th IDHW 2019 included a sub-theme on communication and human behaviour change. This was introduced by a plenary talk by Tamzin Furtado [72], a social scientist with a background in global health and building communities of practice. It was well received by the participants and is to be followed up by a working group led by Tamzin Furtado.

2. BOAS disease recognition and treatment guidelines

Following discussion, it was agreed that there are examples of individual animals in all brachycephalic breeds that appear unaffected by breathing issues despite some assertions to the contrary from some quarters. As a priority, it was agreed that diagnostic criteria were needed that would include the definition of “a healthy dog” in brachycephalic breeds. In addition, guidelines, terminology and defined criteria should be developed and agreed for standardised BOAS diagnostic procedures and follow up of interventions.

Despite the substantial volume of data supporting the negative welfare impact of the BOAS issue, it was concluded that updated definitions for diagnostic criteria for screening and diagnosing BOAS and for decision-making on surgical intervention are necessary. It was proposed to develop a user-friendly BOAS grading system that can be used for screening dogs, guiding breeding, monitoring disease progression and treatment options. Representatives from the Cambridge BOAS group and the University of Surrey agreed to take responsibility for this action.

3. Epidemiology

There are now increasing volumes of population-level data being published on a range of issues relating to brachycephalic health in dogs from research groups such as VetCompass in the UK [91]. It was agreed that open access to the raw data used in these studies could facilitate further analyses of these rich data that could enable even more research questions to be explored and that could benefit dog welfare. Dan O’Neill of the Royal Veterinary College agreed to explore the feasibility of setting up an open access database of raw data within the VetCompass website [91] that could provide such a resource.

4. Obesity

The group agreed that there was now strong evidence supporting associations between obesity and both the occurrence and the severity of BOAS [92, 93]. This suggests that action to limit obesity in brachycephalic breeds could have diverse welfare benefits for these breeds. The Global Pet Obesity Initiative Position Statement calls for adoption of a uniform nomenclature for canine obesity, the adoption of a universal Body Condition Score using a whole-integer, one-through-nine [1–9] scale for dogs and for formal recognition of canine obesity as a disease [94]. Since excess bodyweight is associated with worsening breathing problems in brachycephalic dogs [93, 95], it was concluded that The Global Pet Obesity Initiative Position Statement should be supported. Dan O’Neill of the Royal Veterinary College proposed to the overall plenum that IDHW should formally support the recommendations of the Global Pet Obesity Initiative. There was overwhelming support for the initiative from attendees at the IDHW, and IPFD has now added its name to the list of signatories for the Global Pet Obesity Initiative.

5. Breed standards and education of show judges

Further to discussions at the 3rd IDHW 2017, the need for innovative actions to guide the formulation and interpretation of breed standards and training of show judges for brachycephalic breeds were explored. The absence of representation in the theme from the Fédération Cynologique Internationale (FCI), as well as formally for the American Kennel Club (AKC) limited the discussion on actions taken. The UK Kennel Club (KC) emphasised how importantly it took the brachycephalic issue and stressed its commitment to implementing changes in wording and interpretation of “brachycephalic breed” standards and to making show judges worldwide aware of their responsibility for the entire breed populations [96]. A working group to develop concrete progress on these actions was appointed to be chaired by the UK KC. In a similar manner, the Swedish Kennel Club have initiated the Breed Specific Instructions (BSI), now adopted by the Nordic and many other Kennel Clubs [97].

6. Genetic action plan for extreme morphologies

During discussion on legislation and proposed outcrossing, it was suggested that a genetics action plan for extreme morphologies should be developed that included population and molecular geneticists, as well as a broad spectrum of breeders and breed communities including those already outcrossing. A representative from UC Davis was appointed to head a working group with an extended remit beyond BOAS to other extreme morphologies as well.

7. Creation of an International Brachycephalic Group

The work of the UK Brachycephalic Working Group was held up as evidence that collaboration across all stakeholders can be achieved and maintained for the benefit of dog welfare [98]. It was concluded that an international brachycephalic working group (as an umbrella organisation for a series of national groups across the globe) would be beneficial to identify different approaches and opinions, to share experiences/data and to move forward together, building on experiences from the national initiatives. Monique Megens, with experience from the Federation of Companion Animal Veterinary Associations (FECAVA) and the World Small Animal Veterinary Association (WSAVA), was appointed to lead this action with the help of many other theme participants.

## Discussion

The IDHW is now well established as a recurring key event in the dog welfare calendar. Meetings are spaced at 18–24 month intervals, with this the 4th meeting since the inaugural event in 2012. The meeting structure that has proved effective at previous IDHWs was successfully applied again for the 4th IDHW 2019, with attendees allocated to a specific theme and staying within this group for the duration of the meeting [18]. The 4th IDHW 2019 was structured around 5 key themes. Three of the five themes featured in 2017, namely Breed-specific Health Strategies, Exaggerations and Extremes in Dog Conformation and Genetic Testing for dogs. The Concept of “Breed” and Supply and Demand were new themes for 2019.

The strapline of the IDHW prioritises “Moving from Information and Collaboration to Action”. The review of progress made since the 3rd IDHW 2017 and the comprehensive lists of actions agreed upon during the current meeting suggest that such movement has indeed been achieved. Where the level of progress made had not met expectations, however, this was highlighted and addressed. An example includes the Exaggeration and Extremes theme where it was decided that the general public perception of brachycephalic breeds has not been changed sufficiently. Therefore, a subtheme on communication and human behaviour change and a working group in this specific area has been planned as an action from the theme. Priorities, challenges, opportunities and actions within each theme were determined. Working groups with specific tasks were identified and many plan to continue to communicate through forum communities on DogWellNet.com. As this is the 4th meeting, networks are increasingly well established, which should help in sustaining action into the future and especially to maintain momentum between the meetings.

Participants attending the IDHWs continue to be diverse and there are now well recognised links between different stakeholders, making sustained action possible. In addition, there were participants at the 4th IDHW 2019 who had not attended previously and were able to offer additional knowledge and insights. Despite the diversity of participants, there are groups that could strengthen future discussions. For example, it was noted that the Exaggeration and Extremes theme would have benefitted from attendance by breed health co-ordinators of relevant breed clubs who play a pivotal role in positively influencing breeding decisions and more conformation judges.

### Next meeting

The location and date for the 5th IDHW in 2021 are not yet finalised, but progress towards achieving the action plans specified within each theme at the 4th IDHW 2019 will be presented and reviewed at the 5th IDHW. Dog health and welfare priorities can change over time, therefore new themes are likely to be introduced at the 5th IDHW and new delegates should be encouraged to attend in order to broaden perspectives.

## Conclusions

The IDHW provides a forum for formal and informal discussion between relevant groups so that key dog health and welfare issues can be identified and defined, and plans can be agreed for effective actions to address them. Monitoring of action plans establishes whether progress has been made and evaluates the level of meaningful improvements in canine health and welfare that have been achieved. The 3rd IDHW 2017 resulted in a number of significant outcomes. New and continuing actions were laid down at the 4th IDHW 2019, which will be re-evaluated at the 5th IDHW facilitating continual progress.

## Data Availability

The plenary presentations and speaker biographies used for this paper are available at https://dogwellnet.com/content/ipfd-international-dog-health-workshops/ipfd-international-dog-health-workshop-4/4th-idhw-post-meeting-resources/4th-idhw-theme-presentations-r656/.

## Abbreviations

1st IDHW: 1st International Dog Health Workshop 2012
2nd IDHW: 2nd International Dog Health Workshop 2015
3rd IDHW: 3rd International Dog Health Workshop 2017
4th IDHW: 4th International Dog Health Workshop
5th IDHW: 5th International Dog Health Workshop
AKC: American Kennel Club
AMR: antimicrobial resistance
BHCPs: Breed Health and Conservation Plans
BOAS: brachycephalic obstructive airway syndrome
CGE: Canine Genetics and Epidemiology
FCI: Fédération Cynologique Internationale
GT: genetic testing
GTP: genetic test provider
HGTD: Harmonization of Genetic Testing for Dogs
IPFD: International Partnership for Dogs
ISAG: International Society for Animal Genetics
KC: Kennel Club
